# Reputation-Based Secure Sensor Localization in Wireless Sensor Networks

**DOI:** 10.1155/2014/308341

**Published:** 2014-05-20

**Authors:** Jingsha He, Jing Xu, Xingye Zhu, Yuqiang Zhang, Ting Zhang, Wanqing Fu

**Affiliations:** ^1^School of Software Engineering, Beijing University of Technology, Beijing 100124, China; ^2^College of Computer Science and Technology, Beijing University of Technology, Beijing 100124, China; ^3^Information Center, SINOPEC Research Institute of Petroleum Processing, Beijing 100083, China

## Abstract

Location information of sensor nodes in wireless sensor networks (WSNs) is very important, for it makes information that is collected and reported by the sensor nodes spatially meaningful for applications. Since most current sensor localization schemes rely on location information that is provided by beacon nodes for the regular sensor nodes to locate themselves, the accuracy of localization depends on the accuracy of location information from the beacon nodes. Therefore, the security and reliability of the beacon nodes become critical in the localization of regular sensor nodes. In this paper, we propose a reputation-based security scheme for sensor localization to improve the security and the accuracy of sensor localization in hostile or untrusted environments. In our proposed scheme, the reputation of each beacon node is evaluated based on a reputation evaluation model so that regular sensor nodes can get credible location information from highly reputable beacon nodes to accomplish localization. We also perform a set of simulation experiments to demonstrate the effectiveness of the proposed reputation-based security scheme. And our simulation results show that the proposed security scheme can enhance the security and, hence, improve the accuracy of sensor localization in hostile or untrusted environments.

## 1. Introduction

The technologies of wireless sensor networks (WSNs) are becoming popular along with the rapid advancement of wireless communication technology, more remarkable performance of integrated circuits as well as decrease in cost and increase in functionality of sensor nodes. Since WSNs are a kind of intelligence networks that are able to integrate data collection, fusion, and transmission, such networks have been widely used in fields such as military defense, industrial and agricultural control, urban management, environment monitoring, health care, emergency rescue, and disaster relief. In addition, sensor networks also have a broad prospect of applications in tracking logistics management and space exploration. Depending on different application scenarios in the above areas, researchers have put forward some new technology and strategy, such as sensor deployment methods suitable for underwater detection [1] and intelligent monitoring technologies in Smart Home scenarios [2]. In short, the applications of WSNs are being developed to achieve ubiquity that can bring more convenience for human beings in many areas.

In most applications, sensor nodes are used to collect physical data, such as temperature, humidity, water level, pressure, and wind speed, that are sent along with the location information to the data center to ensure that the collected data have spatial meaning. Furthermore, the location information of sensor nodes can also serve as the basis for some network functions, such as network configuration and real-time statistics of network coverage. Therefore, in massively deployed WSNs, location information of sensor nodes is very important for enabling many applications, which makes sensor localization one of the basic services and a core technology for WSNs.

Since sensor localization in wireless sensor networks (WSNs) is a fundamental technical issue and is critical for monitoring applications and for most location-based routing protocols and services, research in sensor localization technology has generated a wide spread interest and various issues on different aspects have been studied, which include efficiency [3], accuracy [4], and security [5], among many hot issues in sensor localization.

Current algorithms for sensor localization fall into two categories: range-free algorithms [6] and range-based algorithms [7]. In a range-free algorithm, such as Centroid [8] or CTDV-Hop [9], a node estimates its location using information of connectivity between different nodes. In a range-based algorithm, a sensor node estimates its own location based on information about distances or angles between sensor nodes and through using techniques such as time of arrival (TOA) [10], time difference of arrival (TDOA) [11], received signal strength indicator (RSSI) [12], and angle of arrival (AOA) [13] as well as methods such as trilateration, triangulation, or maximum likelihood estimation [14]. Among the many different sensor localization algorithms, RSSI-based positioning technology is perhaps the most popular due to its low cost and easy implementation. On the other hand, sensor localization results can be greatly affected by malicious nodes in hostile or untrusted environments. This is because sensor nodes can hardly perform accurate localization if they use location information that is provided by untrusted beacon nodes. Security in sensor localization has thus received a great deal of attention along with the development of sensor localization technologies for WSNs.

In the past few years, researchers have proposed several security strategies for sensor localization from different aspects. Some of the methods implement verification measures to reduce the impact of using unreliable or false location information [15] while some others apply a series of schemes in which temporal, spatial, and consistent properties are considered to deal with distance-consistent spoofing attacks [16]. However, in these schemes, sensor nodes are divided into just two types: secure and insecure sensor nodes through the mechanisms of comparing the nodes and their behavior against normal situations. However, such an approach cannot be very objective, which could cause many false positive and false negative results.

Meanwhile, some other researchers have proposed localization methods that are able to fight against attacks launched by compromised sensor nodes, a problem that is more difficult to deal with. Liu et al. proposed robust computing algorithms to improve the reliability of localization schemes [17]. Park and Shin proposed an attack-tolerant localization protocol that would perform adaptive management of a profile for normal localization behavior [18]. However, the limitation of these schemes is that they did not consider the security of sensor localization when sensor nodes are joining and leaving the network along with the passage of time. In addition, they did not pay enough attention to secure sensor localization in dynamic wireless networks.

As an effective means of ensuring security, the notion of reputation has been introduced and some reputation-based schemes have since been proposed for sensor localization. Srinivasan et al. proposed a distributed reputation-based beacon trust system [19] and Xu et al. proposed a reputation-based revising scheme for sensor localization which would incur high computation cost [20]. However, complicated reputation evaluation in the above schemes for sensor localization makes it necessary to further improve the efficiency of evaluation for beacon nodes. Any sensor localization method that can achieve good performance should ensure the reliability of location information before such information can be actually used for sensor localization.

In real applications, there may be other types of sensor localization methods to fit different application scenarios. Therefore, specific localization methods in real applications need to be continuously developed and improved based on orientation methods in order to adapt basic sensor localization schemes to the many different network scenarios. Consequently, in order to develop effective sensor localization methods, we should analyze and understand the main characteristics of specific networks and develop proper performance metrics that can be used to measure the performance of sensor localization schemes. In addition, we should also consider limitations of wireless sensor networks such as constrained energy supply in the sensor nodes as well as the complexity of network environments in the development of effective sensor localization methods.

In this paper, we propose a novel reputation-based secure sensor localization scheme to improve the accuracy of sensor localization for WSNs in hostile environments. In the proposed reputation model, the reputation of each beacon node is evaluated by each other to ensure that sensor nodes will get credible location information to perform sensor localization. The proposed scheme can therefore effectively reduce the impact of malicious beacon nodes on the localization of regular sensor nodes by relying on the security mechanism of beacon node evaluation. Our simulation results show that the proposed reputation-based secure sensor localization scheme can improve the accuracy of sensor localization in hostile or untrusted environments. In addition, the proposed secure sensor localization scheme possesses the desirable characteristics of expandability and flexibility since it can be used in both static and dynamic networks.

The remainder of this paper is structured as follows. In Section 2, we present a reputation model in which we first describe the network model and then propose a reputation evaluation model. In Section 3, we present our sensor localization scheme which is based on the evaluation of the reputation of beacon nodes. In Section 4, we describe the simulation that we have performed and present the simulation results. Finally, in Section 5, we conclude this paper in which we also discuss some future work.

## 2. The Reputation Model

In hostile network environments, which most current WSN deployments would assume, regular sensor nodes need to be confronted with security threats during the process of sensor localization. If a sensor node can identify the security and credibility of location information that it receives and subsequently use the information appropriately, the accuracy of sensor localization can be greatly improved or ensured in such environments. Therefore, in order to develop effective sensor localization schemes, we should understand the main characteristics of the specific networks as well as the performance goals of the localization schemes. To achieve the above objective, we need to consider such characteristics as resource constraints in the sensor nodes and the complexity of the environment where the sensor nodes are deployed. Any sensor localization scheme must be effectively working in a specific WSN after the above-mentioned factors are considered in the design.

To achieve the above goal, we first propose a reputation scheme to be used in the sensor localization scheme we will propose later in this paper to deal with a hostile deployment environment in which malicious nodes can be dropped into the network at will and regular sensor nodes can also be easily compromised to make them behave in a malicious manner. We call the scheme that we propose the reputation-based localization scheme (RBL). The main characteristics of the reputation model and the RBL are as follows.

1. The proposed secure sensor localization scheme is developed based on a reputation model and on the evaluation of all the beacon nodes for deriving a reputation value for each and every beacon node.
2. In the reputation model, the reputation of each beacon node is evaluated and consequently used by regular sensor nodes to determine the credibility of the location information provided by the beacon node.
3. In the reputation model, the reputation of each beacon node is updated continuously with the passage of time if sensor localization needs to be carried out from time to time.

In the following sections, we will first describe the network model followed by the threat model and the reputation model.

### 2.1. The Network Model

The WSN under consideration is composed of beacon nodes and regular sensor nodes. Beacon nodes are capable of positioning themselves (e.g., by determining their positions through GPS) while the regular sensor nodes need to locate their own positions based on position information from other nodes, especially from the beacon nodes.

Our sensor localization method in this paper requires that a regular sensor node first estimates its relative position to some of the beacon nodes through the means of receiving signals from creditable beacon nodes and by computing the distances between them using a signal attenuation formula. Then, the sensor node estimates its position using the maximum likelihood estimation method [21] after it has collected enough position information.

### 2.2. The Threat Model

An analysis of the network model described above indicates that position information received from beacon nodes and the estimation of relative positions between a regular sensor node and the referenced beacon nodes can determine the accuracy of sensor localization. There are, however, two primary types of security threats for the network model as described below.

**Figure 1:**
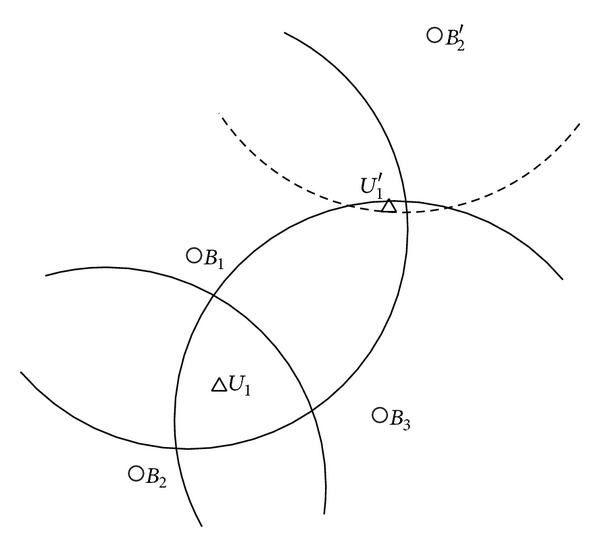
An example of sensor localization in hostile environments.

1. Sending false beacon information: if malicious beacon nodes send false position information, such information received by regular sensor nodes may not be accurate. Then, the estimated position of a regular sensor node cannot be guaranteed to be accurate and will lose its credibility because it is calculated based on received false information from beacon nodes. The impact to sensor localization from this type of attacks is shown in Figure 1. We can see from the figure that *B*_2_′ is the false position of beacon node *B*_2_, which makes sensor node *U*_1_ receive a false localization result *U*_1_′.
2. Obstructing physical property: if malicious nodes interfere with normal signals from beacon nodes, no regular sensor node would be able to estimate its relative position to the beacon nodes accurately by the means of signal attenuation, leading to reduced accuracy for sensor localization.

Consequently, the scheme that we propose in this paper needs to deal with the potential threats that result from the above two types of attacks in order to improve the accuracy of sensor localization in WSNs.

### 2.3. The Proposed Reputation Model

To deal with the above security threats, we propose a novel reputation model for sensor localization in WSNs. In the reputation model, beacon nodes evaluate each other using information such as the characteristics about the perception of positions and provide the evaluation results to the regular sensor nodes. The regular sensor nodes use the evaluation results provided by the beacon nodes to rank the beacon nodes and base the credibility of the location information provided by beacon nodes on such ranking.

First, let us make the following assumptions in our reputation model.

1. The reputation value for each and every beacon node is a number between 0 and 1, indicating values from the lowest to the highest reputations.
2. The reputation value for each and every beacon node is initialized to be 0.5, a medium value to start with.
3. In the reputation model, each beacon node performs evaluation only on its neighboring beacon nodes, that is, the beacon nodes that are one hop away from it.

Pseudocode 1 contains the pseudocode for our proposed reputation model for beacon node *B*_*j*_ and sensor node *U*_*m*_.

**Pseudocode 1:**
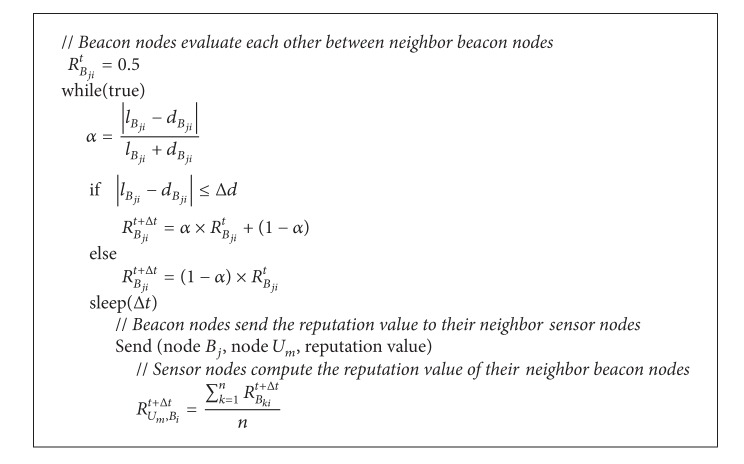
Pseudocode for the reputation model.

The details of the evaluation procedure in the proposed reputation model are as follows.

(1) Beacon node *B*_*i*_ sends its coordinate (*x*_*i*_, *y*_*i*_) to its neighboring beacon nodes.
(2) Each neighboring beacon node to *B*_*i*_ will calculate its distance to *B*_*i*_ using the received coordinate information and the signal strength information independently. Let *l*_*B*_*ji*__ denote the distance between *B*_*j*_ and *B*_*i*_ based on the coordinate information and let *d*_*B*_*ji*__ denote the distance based on the signal strength information. *B*_*j*_ can then calculate *l*_*B*_*ji*__ using the coordinate information from *B*_*i*_ and calculate *d*_*B*_*ji*__ through a signal strength ranging algorithm based on the strength of the signals received from *B*_*i*_.
(3) All the neighboring beacon nodes evaluate the reputation of *B*_*i*_. The value of reputation evaluation is determined using (1) in which *R*_*B*_*ji*__^*t*^ and *R*_*B*_*ji*__^*t*+Δ*t*^ denote the reputation values on *B*_*i*_ by *B*_*j*_ at times *t* and *t* + Δ*t*, respectively, and Δ*t* denotes the time interval of two reputation values. Let Δ*d* be the threshold for the distance, that is, the error that can be tolerated for the distance, and let *α* be the weight of the evaluation value which is determined using (2)

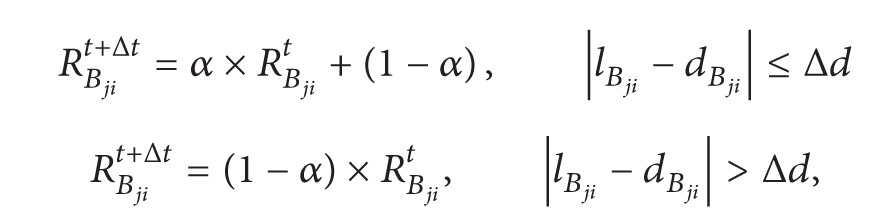
(1)

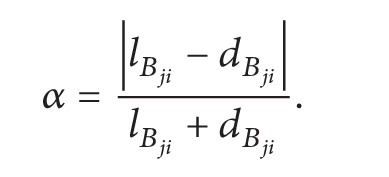
(2)
(4) The neighboring sensor nodes get the reputation values for *B*_*i*_ from the beacon nodes. Each regular sensor node collects the evaluation values from all the neighboring beacon nodes and computes the average reputation value using (3) in which *R*_*U*_*m*_,*B*_*i*__^*t*+Δ*t*^ and *R*_*B*_*ki*__^*t*+Δ*t*^ denote the reputation value on beacon node *B*_*i*_ from a sensor node *U*_*m*_ and that on *B*_*i*_ evaluated by *B*_*k*_ at time *t* + Δ*t*, where *B*_*k*_ is a neighboring beacon node to *B*_*i*_ and *n* is the number of such neighboring nodes. Consider
  (3) $$\begin{array}{c} R_{U_{m},B_{i}}^{t+\Delta t}=\frac{\sum_{k=1}^{n}R_{B_{ki}}^{t+\Delta t}}{n}. \end{array}$$
(5) Every regular sensor node ranks the neighboring beacon nodes from high to low based on the received reputation values.

## 3. The Sensor Localization Scheme

The sensor localization scheme in this paper uses the proposed reputation evaluation scheme described above in which the reputation model is relied upon by the beacon nodes to evaluate each other. In the illustration below, we use the RSSI ranging technology for sensor localization although the same reputation scheme can be applied equally to TOA, TDOA, and AOA ranging methods in practical applications.

After receiving the evaluation results, a regular sensor node will select credible beacon nodes based on the reputation values. Afterwards, the sensor node will measure the distance to the credible beacon nodes using the RSSI ranging technology and estimate its location through maximum likelihood estimation. The main steps in our localization scheme are described as follows.

**Figure 2:**
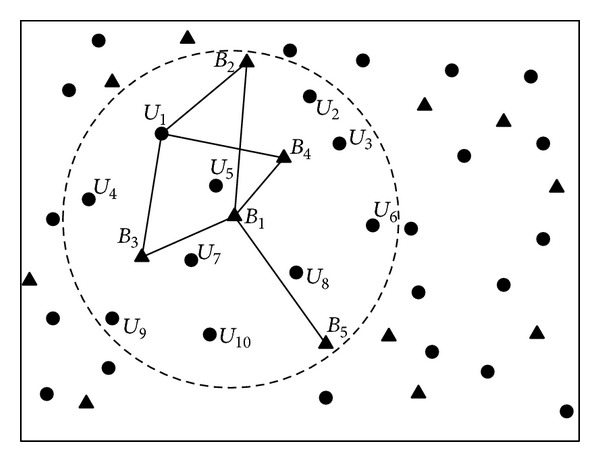
The network topology.

(i) Every beacon node provides its location information to all the neighbor nodes. As shown in Figure 2, beacon node *B*_1_ sends its position coordinate (*x*_1_, *y*_1_) to all the neighboring nodes.
(ii) Beacon nodes in the network will evaluate each other using the proposed reputation model and each will send its evaluation results to all the neighboring nodes. As shown in Figure 2, beacon nodes *B*_2_, *B*_3_, *B*_4_, and *B*_5_ evaluate the reputation of *B*_1_ after receiving the location information from *B*_1_ using the proposed reputation model and each will send the evaluation result to their neighboring sensor nodes including node *U*_1_.
(iii) Each regular sensor node will select credible beacon nodes based on the results from the reputation evaluation. Sensor node *U*_1_ computes the reputation value for *B*_1_ and collects the reputation values from neighboring beacon nodes using (3). Then, *U*_1_ ranks the neighboring beacon nodes according to the reputation values in the order of high to low, based on which it selects the credible beacon nodes accordingly.
(iv) Regular sensor nodes estimate their relative positions to the credible beacon nodes using the signal attenuation formula in RRSI [12].
(v) Regular sensor nodes calculate their coordinates using maximum likelihood estimation. Suppose that the number of credible neighboring beacon nodes around *U*_1_ is *p* with coordinates (*x*_1_, *y*_1_), (*x*_2_, *y*_2_),…, (*x*_*p*_, *y*_*p*_), respectively, and the distances between *U*_1_(*x*_*U*_1__, *y*_*U*_1__) and the beacon nodes are *d*_1_, *d*_2_,…, *d*_*p*_, respectively; then the position of *U*_1_ can be calculated using the following:
  (4) $$\begin{array}{c} {\left(x_{U_{1}}-x_{i}\right)}^{2}+{\left(y_{U_{1}}-y_{i}\right)}^{2}=d_{i}^{2},\;i=1,2,\ldots ,p. \end{array}$$

In addition, *p* distance equations about *U*_1_ and the *p* beacon nodes are listed as in (5) that result from subtracting the last equation from each of the first *p* − 1 equations. Consider

(5) x12−xp2−2(x1−xp)xU1+y12−yp2 −2(y1−yp)yU1=d12−dp2⋯xp−12−xp2−2(xp−1−xp)xU1+yp−12−yp2 −2(yp−1−yp)yU1=dp−12−dp2.

*U*_1_(*x*_*U*_1__, *y*_*U*_1__) can then be calculated using the following:

(6) $$\begin{array}{c} U_{1}=A^{-1}b. \end{array}$$

The matrices in (6) can then be expressed as the following expressions:

(7) $$\begin{array}{c} A=2\left[\begin{array}{cc} x_{1}-x_{p} & y_{1}-y_{p}\\ \cdots & \\ x_{p-1}-x_{p} & y_{p-1}-y_{p} \end{array}\right],\\ b=\left[\begin{array}{c} x_{1}^{2}-x_{p}^{2}+y_{1}^{2}-y_{p}^{2}-d_{1}^{2}+d_{p}^{2}\\ \cdots \\ x_{p-1}^{2}-x_{p}^{2}+y_{p-1}^{2}-y_{p}^{2}-d_{p-1}^{2}+d_{p}^{2} \end{array}\right],\\ U_{1}=\left[\begin{array}{c} x_{U_{1}}\\ y_{U_{1}} \end{array}\right]. \end{array}$$

The final solution to (6) can be obtained using the following:

(8) $$\begin{array}{c} U={\left(A^{T}A\right)}^{-1}A^{T}b. \end{array}$$

From the above steps, we can see that a regular sensor node would treat the location information from neighboring beacon nodes differently according to the result of reputation evaluation. There is no need to determine the position relationship between regular sensor nodes and beacon nodes that have low reputation values, which are required in the signal attenuation formula, resulting in reducing a certain amount of computational overhead.

## 4. Simulation and Analysis

We have performed some simulation on wireless sensors localization with the proposed RBL to evaluate the performance of the scheme.

The network configuration for the simulation is set up as follows. The regular sensor nodes and beacon nodes are deployed randomly in an area of 650 m × 600 m. The transmission radius of each beacon and sensor node is set at 200 m. There exist some malicious beacon nodes that randomly send out false location information.

Localization error is one important indicator of the performance in sensor localization for WSNs, which is calculated using (9). In the formula, (*x*_*U*_*m*__, *y*_*U*_*m*__) and (*x*_*U*_*m*__′, *y*_*U*_*m*__′) denote the measured coordinates and the actual coordinates for node *U*_*m*_, respectively, *R* denotes the transmission radius of the nodes, and *e*_*m*_ is the localization error. Consider

(9) $$\begin{array}{c} e_{m}=\frac{\sqrt{{\left(x_{U_{m}}-x_{U_{m}}^{\prime }\right)}^{2}+{\left(y_{U_{m}}-y_{U_{m}}^{\prime }\right)}^{2}}}{R}. \end{array}$$

**Figure 3:**
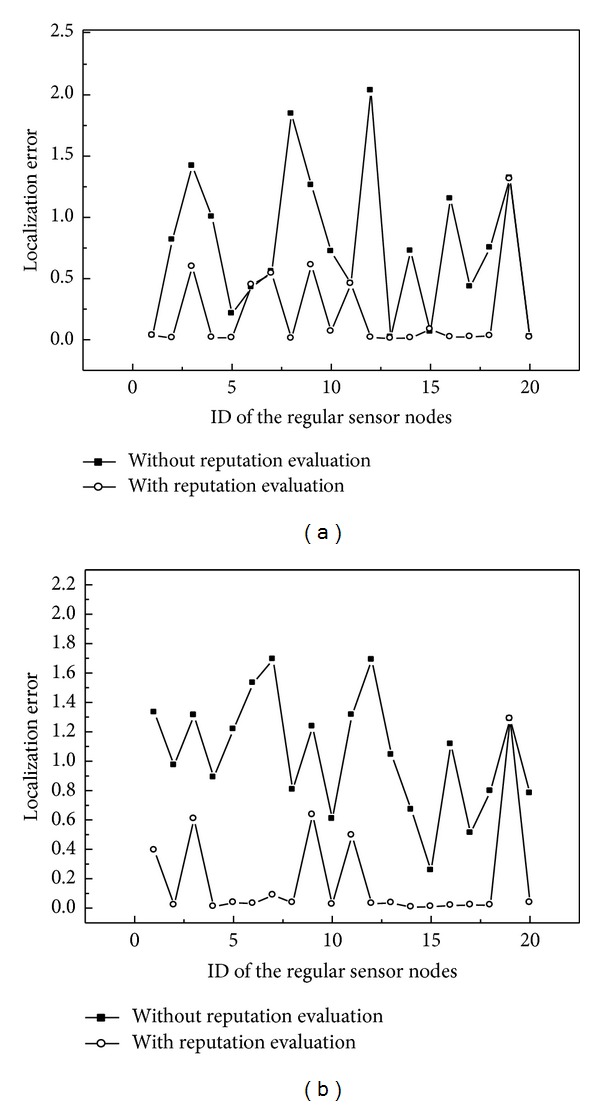
Sensor localization error with a different number of malicious beacon nodes: (a) 10 normal and 5 malicious beacon nodes; (b) 10 normal and 10 malicious beacon nodes.

The localization error from the simulation for 20 sensor nodes is shown in Figure 3. We can see from the figure that reputation evaluation is effective for reducing localization error in hostile environments and the improvement is more significant as the number of malicious beacon nodes increases.

**Figure 4:**
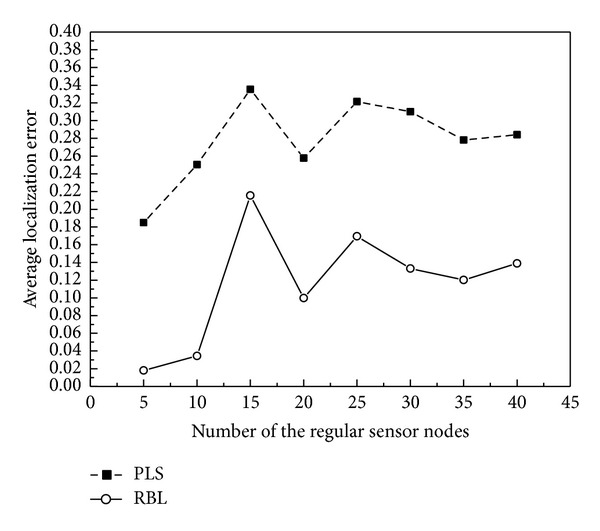
Average sensor localization error with a varying number of regular sensor nodes.

There are two types of threats in sensor localization: attacks targeted at the nodes and attacks targeted at the location information. RBL evaluates the credibility of beacon nodes by evaluating the location information that beacon nodes provide in order to reduce the influence of compromised beacon nodes on localization results and to resist the threat of location information tampering by the malicious beacon nodes. To measure the capability of RBL on countering the above security threats, in our evaluation, we deploy 40 regular sensor nodes to expand the scale of our experiment in which we measure the average localization error using (10) where *N* denotes the number of regular sensor nodes in the network. The average localization error from our simulation on a network in which there exist one or more compromised beacon nodes is shown in Figure 4. We can see from the figure that although the average localization error fluctuates with the number and the locations of the regular sensor nodes, the result of RBL is much better than that of the primary localization scheme (PLS) using RRSI in which no evaluation of beacon nodes is performed. Consider

(10) $$\begin{array}{c} \overset{-}{e}=\frac{\sum_{i=1}^{N}e_{i}}{N}. \end{array}$$

Since WSNs possess the characteristics of dynamic network topology, an advanced secure sensor localization scheme should not only be able to ensure the security of sensor localization in a static network, but also be able to handle the cases of nodes joining, leaving, and removing from the network. We have performed some simulations on sensor localization for the above scenarios.

In order to expand the coverage of beacon nodes in a network so as to make more regular sensor nodes the neighbors of the beacon nodes in the network, consequently improving the utilization of beacon information, we can increase the signal transmission power of the beacon nodes to effectively expand the signal transmission radius of the beacon nodes.

**Figure 5:**
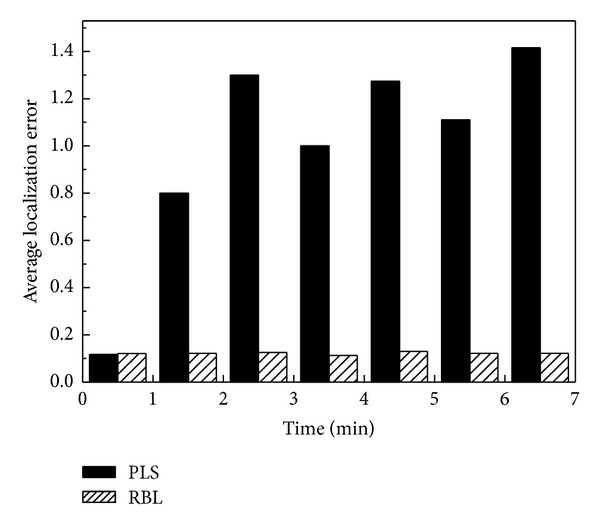
Average sensor localization error as beacon nodes are added into the network.

In the simulations, we first deploy 20 regular nodes and 4 normal beacon nodes in the area. Then, we add more beacon nodes into the network at the rate of one node per minute starting at the moment of 1.5 min with normal and malicious beacon nodes being added alternately. Malicious beacon nodes that are added into the network would send out false position information randomly while normal beacon nodes always send out their real position information.  Figure 5 shows the average localization error for regular sensor nodes during the first seven minutes from which we can see that the average localization error for regular sensor nodes fluctuates noticeably in the primary localization scheme but exhibits a good performance in our proposed RBL.

**Figure 6:**
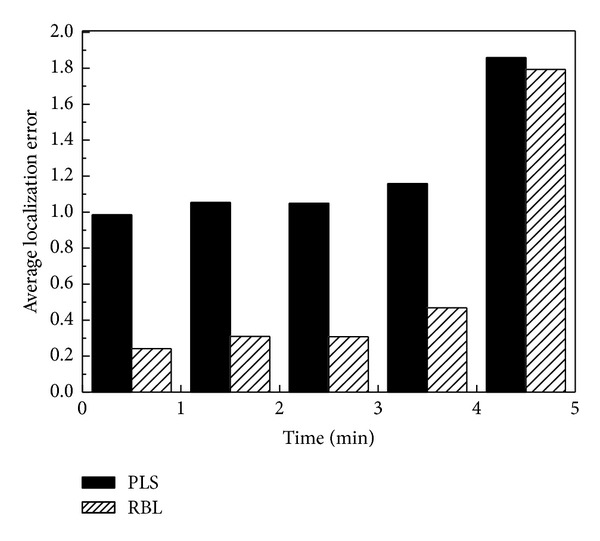
Average sensor localization error as beacon nodes are removed from the network.

We have also performed some simulations to evaluate the impact of nodes leaving the network on sensor localization. In the simulation, we first deploy 20 regular nodes, 4 normal beacon nodes, and 4 malicious beacon nodes in the area. Then, we remove the beacon nodes from the network at the rate of one node per 1 minute starting at the moment of 1.5 min with normal and malicious beacon nodes being removed alternately. Again, normal beacon nodes always claim their real positions while malicious beacon nodes would send out false position information randomly.  Figure 6 shows the average sensor localization error for regular sensor nodes during the process from which we can see that the average localization error of RBL is noticeably lower than that of PLS in most cases. However, when the number of normal beacon nodes falls below three in the whole network, the advantage would disappear, which seems to be a limitation of the current RBL.

Lastly, we evaluate the impact of status change among the existing beacon nodes on regular sensor nodes under the assumption that the total number of beacon nodes remains the same. Four possibilities exist for such status change as illustrated in Table 1 in which *RP*_1_ and *RP*_2_ represent the cases in which a beacon node does not change its real position and changes its real position, respectively, and *CP*_1_, *CP*_2_, and *CP*_3_ represent the cases in which a beacon node does not change its claimed position information, changes its claimed position information randomly, and changes its claimed position information consistently, respectively.

We perform evaluations for four scenarios. In the first evaluation, we deploy 20 regular sensor nodes and 8 normal beacon nodes in the area and then change the status of 4 beacon nodes to the state that corresponds to situation 1 in Table 1 gradually during a 4-minute time period starting at the moment of 1.5 min. In the second evaluation, we deploy 20 regular sensor nodes and 8 normal beacon nodes in the area and then change the status of 4 beacon nodes to the state that corresponds to situation 2 in Table 1 gradually during a 4-minute time period starting at the moment of 1.5 min. In the third evaluation, we deploy 20 regular sensor nodes and 8 normal beacon nodes in the area and then change the status of 4 beacon nodes to the state that corresponds to situation 3 in Table 1 gradually during a 4-minute time period starting at the moment of 1.5 min. In the last evaluation, we deploy 20 regular sensor nodes and 8 normal beacon nodes in the area and then change the status of 4 beacon nodes to the state that corresponds to situation 4 in Table 1 gradually during a 4-minute time period starting at the moment of 1.5 min.

**Figure 7:**
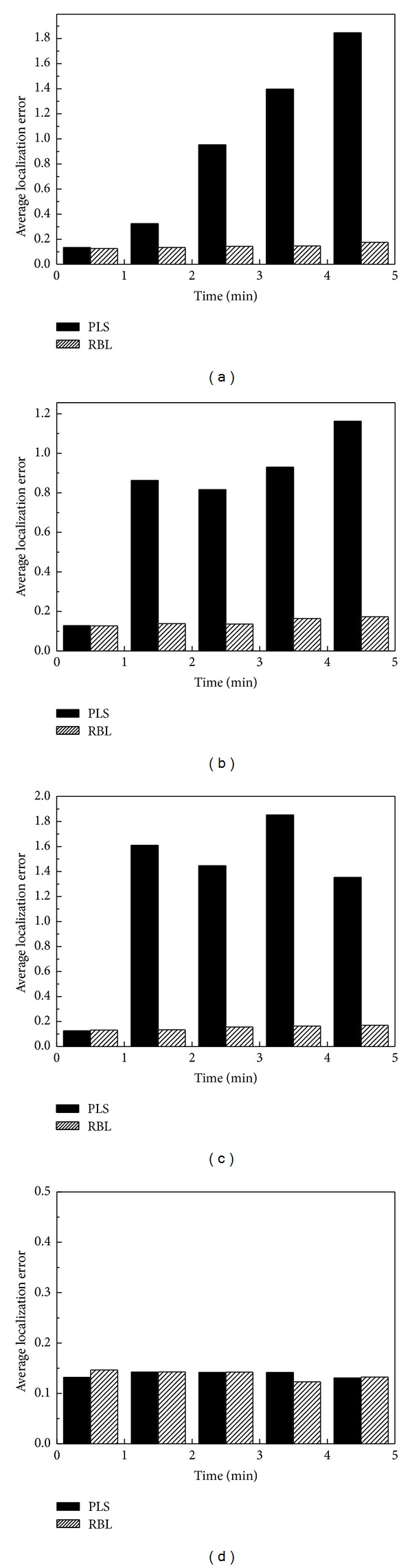
Average sensor localization error as beacon nodes change their status in various scenarios.

Figures 7(a), 7(b), 7(c), and 7(d) show the results of the evaluations that correspond to the above four evaluation scenarios. We can see from the figure that RBL can effectively filter out abnormal (or malicious) beacon nodes when some of the beacon nodes change their status in an unpredictable manner, which demonstrates that RBL is an effective scheme for secure sensor localization for WSNs, which clearly shows that RBL can improve the accuracy of sensor localization in hostile or untrusted environments.

In summary, so far, we have performed three sets of simulation experiments to verify the performance and the effectiveness of the proposed security scheme for sensor localization in hostile or untrusted environments. In the first one, we evaluated the performance of localizing regular sensor nodes in the presence of a varying number of malicious beacon nodes. In the second one, we evaluated the average sensor localization error for different numbers of regular sensor nodes in hostile or untrusted environment. In the third one, we evaluated sensor localization results; when new beacon nodes join the network, existing beacon nodes leave the network and existing beacon nodes change their status that determines how they would make claims on their positions. It is clear that the purpose of the last experiment is to evaluate the influence on the localization of regular sensor nodes due to changes on the credibility of the beacon nodes. That is, the first two experiments are mainly aimed at showing the performance of RBL on secure sensor localization in static WSNs while the third one is aimed at verifying the effectiveness of RBL on secure sensor localization in dynamic WSNs. All the simulation results that we have obtained clearly show that RBL can reduce the effect of malicious beacon nodes on the localization of regular sensor node, thus allowing us to conclude that RBL can effectively improve the security and the accuracy of sensor localization in WSNs. The experiments also indicate that RBL can scale well with the size of the network and can be applied in dynamic WSNs, especially when new sensor nodes can join and existing sensor nodes can leave networks with the passage of time.

## 5. Conclusion

In this paper, we proposed a novel reputation model for regular sensor nodes to evaluate the credibility of beacon nodes in sensor localization. In the model, beacon nodes first evaluate each other and then provide the evaluation results to regular sensor nodes for them to determine the credibility of beacon nodes to ensure that they will receive and use credible position information from the beacon nodes in locating their own positions. The proposed security scheme can improve the accuracy of sensor localization in hostile or untrusted environments. The scheme can help to ensure the reliability of received location information under the scenario of signal attenuation by minimizing the effects of false location information as well as interfering signals caused by malicious beacon nodes.

In the future, we will extend our security scheme to counter other types of malicious attacks in sensor localization without incurring too much additional computational cost and communication overhead and to apply our reputation-based sensor localization scheme to different network environments to further verify and improve the scheme. We will also study the impact on evaluation due to other factors of sensor nodes to further improve the performance and usability of our secure sensor localization scheme in WSNs.

## Figures and Tables

**Table 1:** State situations for the beacon nodes.

Categories	1	2	3	4

Situations	*RP*_1_∩*CP*_2_	*RP*_2_∩*CP*_1_	*RP*_2_∩*CP*_2_	*RP*_2_∩*CP*_3_

## Notations

*B*_*i*_: Beacon node *i*
*U*_*m*_: Sensor node *m*
Δ*t*: The time interval of the two reputation values
*l*_*B*_*ji*__: The distance between beacon node *j* and *i* based on the coordinate information
*d*_*B*_*ji*__: The distance between beacon node *j* and *i* based on the ranging techniques (such as received signal strength indicator)
*R*_*B*_*ki*__^*t*^: The reputation value on beacon node *i* from a beacon node *k*
*R*_*U*_*m*_,*B*_*i*__^*t*^: The reputation value on beacon node *i* from a sensor node *m* at time *t*
(*x*_*p*_, *y*_*p*_): The coordinate of beacon node *p*
(*x*_*U*_*m*__, *y*_*U*_*m*__): The coordinate of sensor node *m*.

## References

[1] Golen EF, Yuan B, Shenoy N (2009). An evolutionary approach to underwater sensor deployment. *International Journal of Computational Intelligence Systems*.

[2] Augusto JC, Liu J, McCullagh P, Wang H, Yang J-B (2008). Management of uncertainty and spatio-temporal aspects for monitoring and diagnosis in a smart home. *International Journal of Computational Intelligence Systems*.

[3] Yang S-K, Ssu K-F (2009). An energy efficient protocol for target localization in wireless sensor networks. *World Academy of Science, Engineering and Technology*.

[4] Boushaba M, Hafid A, Benslimane A (2009). High accuracy localization method using AoA in sensor networks. *Computer Networks*.

[5] Sugihara R, Gupta RK Sensor localization with deterministic accuracy guarantee.

[6] Park J, Lim Y, Lee K, Choi Y-H (2011). A polygonal method for ranging-based localization in an indoor wireless sensor network. *Wireless Personal Communications*.

[7] Chan YWE, Soong BH (2011). A new lower bound on range-free localization algorithms in wireless sensor networks. *IEEE Communications Letters*.

[8] Bulusu N, Heidemann J, Estrin D (2000). GPS-less low-cost outdoor localization for very small devices. *IEEE Personal Communications*.

[9] Wu H, Deng M, Xiao L, Wei W, Gao A (2011). Cosine theorem-based DV-hop localization algorithm in wireless sensor networks. *Information Technology Journal*.

[10] Güvenç I, Chong C-C (2009). A survey on TOA based wireless localization and NLOS mitigation techniques. *IEEE Communications Surveys and Tutorials*.

[11] Savvides A, Han C-C, Strivastava MB Dynamic fine-grained localization in ad-hoc networks of sensors.

[12] Bahl P, Padmanabhan VN RADAR: an in-building RF-based user location and tracking system.

[13] Niculescu D, Nath B Ad hoc positioning system (APS) using AOA.

[14] Zhang Y, Bao L, Yang S-H, Welling M, Wu D (2010). Localization algorithms for wireless sensor retrieval. *Computer Journal*.

[15] Čapkun S, Rasmussen KB, Čagalj M, Srivastava M (2008). Secure location verification with hidden and mobile base stations. *IEEE Transactions on Mobile Computing*.

[16] Chen H, Lou W, Wang Z (2010). A novel secure localization approach in wireless sensor networks. *EURASIP Journal on Wireless Communications and Networking*.

[17] Liu D, Ning P, Liu A, Wang C, Du WK (2008). Attack-resistant location estimation in wireless sensor networks. *ACM Transactions on Information and System Security*.

[18] Park T, Shin KG (2008). Attack-tolerant localization via iterative verification of locations in sensor networks. *Transactions on Embedded Computing Systems*.

[19] Srinivasan A, Teitelbaum J, Jie W DRBTS: distributed reputation-based beacon trust system.

[20] Xu X, Jiang H, Huang L, Xu H, Xiao M A reputation-based revising scheme for localization in wireless sensor networks.

[21] Rusnac R-I, Gontean AŞ Maximum Likelihood Estimation Algorithm evaluation for wireless sensor networks.

